# 口服长春瑞滨节拍化疗治疗晚期多线治疗失败的非小细胞肺癌的临床观察

**DOI:** 10.3779/j.issn.1009-3419.2017.11.03

**Published:** 2017-11-20

**Authors:** 舒洋 姚, 艳斐 顾, 毅 张

**Affiliations:** 1 100053 北京，首都医科大学宣武医院胸外科 Department of Thoracic Surgery, Xuan Wu Hospital, Capital Medical University, Beijing 100053, China; 2 100015 北京，北京和睦家医院 Beijing United Family Healthcare Hospital, Beijing 100015, China

**Keywords:** 肺肿瘤, 长春瑞滨, 节拍化疗, 疗效, 安全性, Lung neoplasms, Vinorelbinen, Metronomic chemotherapy, Efficacy, Safety

## Abstract

**背景与目的:**

晚期非小细胞肺癌（non-small cell lung cancer, NSCLC）患者接受新的多线治疗有助于延长患者的总生存时间。节拍化疗通过多种机制控制肿瘤生长，而且副作用更小。口服长春瑞滨是进行节拍化疗的合适药物。因此，我们对口服长春瑞滨节拍化疗治疗晚期多线治疗失败的NSCLC的疗效及不良反应进行了分析。

**方法:**

收集2016年3月-2017年1月北京和睦家医院及首都医科大学宣武医院收治的26例接受口服长春瑞滨节拍化疗的晚期多线治疗失败的NSCLC的临床资料，并进行回顾性分析。

**结果:**

中位随访时间为4（2-12）个月。患者的中位治疗周期数为2（1-8）个，无患者达到完全缓解，2例（8%）患者达部分缓解，11例（42%）达疾病稳定，13例（50%）为疾病进展。患者的有效率为8%，疾病控制率为50%。中位疾病无进展时间（progression-free survival, PFS）为2.0个月。对影响PFS的各因素进行单因素分析中，体能状态（performance status, PS）评分为1分的患者优于2分患者（*P*=0.012）。PFS与性别、年龄、吸烟状态和病理类型均无关。治疗的耐受性好，严重毒性反应非常少见。没有出现Ⅳ级或不可耐受的毒性反应。没有患者因不良反应（adverse events, AEs）出现死亡或因治疗AEs而需要住院治疗。

**结论:**

口服长春瑞滨节拍化疗可作为治疗晚期NSCLC，尤其是PS评分差的患者的有效药物，安全性高，患者的耐受性好。

## Abstract (en)

**Background and objective:**

The development of new treatments beyond first-line in metastatic non-small cell lung cancer (NSCLC) contributed to the increase in overall survival. Metronomic chemotherapy involves several mechanisms of anti-tumor with less toxicity. Oral vinorelbine has paved the way for innovative treatment strategies through metronomic regimens. Therefore, this study assessed the efficacy and safety of metronomic oral vinorelbinen in advanced NSCLC after failure to multiple-lines treatments.

**Methods:**

Our retrospective study enrolled 26 patients who received metronomic oral vinorelbinen. Survival factors were evaluated by univariate regression analysis.

**Results:**

The median follow-up time was 4 months (range 2-12). The median number of treatment cycles was 2 (range 1-8). No patient achieved complete remission, 2 cases (8%) partial remission, 11 cases (42%) stable disease, 13 cases (50%) progression disease. Overall response rate was 8% and disease control rate was 50%. The median progression-free survival (PFS) was 2 months. In univariate analysis, patients with performance status (PS)=1 had a statistically significantly longer PFS than patients with PS=2. Gender, age, smoking status and histology were not prognostic factors according to PFS. Treatment was well tolerated with rare serious toxicity. No grade 4 adverse events (AEs) or occurrences of intolerable toxicity were observed. There was no treatment-related death and none of the study patients required hospitalization for treatment-related adverse events.

**Conclusion:**

Metronomic oral vinorelbinen is effective in advanced NSCLC after the failure of multiple lines treatments with an acceptable AE profile, especially in patients with high PS.

非小细胞肺癌（non-small cell lung cancer, NSCLC）仍是世界范围内发病率和死亡率最高的恶性肿瘤^[1]^。随着分子生物学和转化医学的快速发展，针对肿瘤驱动基因的靶向药物已逐渐成为晚期NSCLC的标准治疗方案^[2-4]^。而对于没有敏感突变基因的NSCLC患者来说，化疗仍是这部分患者延长生存时间、改善生活质量的关键。而传统的化疗是通过给予最大耐受剂量（maximum tolerated dose, MTD）来达到最佳疗效，毒性反应大，患者耐受性差。约50%的一线化疗进展患者会因体能状态（performance status, PS）不佳，无法耐受二线化疗^[5]^。

节拍化疗是规律给予低剂量的细胞毒药物，患者耐受性更好，治疗的总剂量能达到更高，尤其适用于高龄或体弱的患者^[6]^。节拍化疗的药代动力学能使肿瘤细胞持续暴露在细胞毒药物中，能防止肿瘤细胞出现传统化疗两次治疗间歇期中发生的再次生长。同时，化疗的毒性反应可因降低血药峰浓度而大大降低。此外，节拍化疗还能通过其他机制来发挥抗肿瘤作用，如抗血管生成：靶向作用于肿瘤血管，并且通过抑制调节性T细胞以及诱导树突细胞成熟来加强抗肿瘤的免疫反应^[7]^。

长春瑞滨是一种半合成的长春花生物碱，可选择性作用于微管蛋白，使肿瘤细胞在有丝分裂过程中微管形成障碍。由于它良好的疗效和可控的毒性反应，它是治疗NSCLC化疗药物中的主力军。长春瑞滨口服剂型的生物利用度为33%-43%^[8]^，其细胞毒活性与静脉剂型相似^[9]^。长春瑞滨口服剂型患者在家可自行服用，是进行节拍化疗的合适药物。现对北京和睦家医院和宣武医院2016年3月-2017年1月收治的26例接受口服长春瑞滨节拍化疗的晚期多线治疗失败的NSCLC的临床资料进行回顾性分析，探讨疗效及安全性。

## 1 材料与方法

### 1.1 研究对象

入选标准：①北京和睦家医院或首都医科大学宣武医院经病理或细胞学确诊的晚期NSCLC患者；②影像学资料提示既往治疗中进展或治疗后复发；③至少接受1个月的口服长春瑞滨化疗；④PS评分1分-2分。排除标准：①不适合接受化疗的患者；②预计生存时间 < 3个月；③PS评分 > 2分；④血清总胆红素≥1.5倍正常值的患者；⑤中性粒细胞计数绝对值≤1, 500/μL的患者。

选取2016年3月-2017年1月收治的26例接受口服长春瑞滨节拍化疗的晚期NSCLC患者，所有患者均签署知情同意书。

### 1.2 治疗方法

长春瑞滨（皮尔法伯公司，诺维本），每周一、周三、周五口服30 mg，每28天为1周期，至疾病进展或不良反应不能耐受时停药。

### 1.3 观察指标及评价标准

患者每2个周期或因症状明显加剧进行计算机断层扫描（computed tomography, CT）、磁共振成像（magnetic resonance imaging, MRI）等相关影像学检查评价。按照实体肿瘤疗效评价标准评价疗效，分为完全缓解（complete remission, CR）、部分缓解（partial remission, PR）、疾病稳定（stable disease, SD）及疾病进展（progressive disease, PD）。以CR+PR计算总有效率（overall response rate, ORR），CR+PR+SD计算疾病控制率（disease control rate, DCR）。无进展生存时间（progression-free survival, PFS）定义为从接受口服长春瑞滨节拍化疗开始至疾病进展的时间或死亡时间。药物不良反应（adverse event, AE）：根据常用药物毒性标准（Common Toxicity Criteria, CTC）AE V3.0版^[10]^制定的药物不良反应进行分级。

### 1.4 随访

采用门诊或电话方式随访，末次随访时间为2017年5月15日。从口服长春瑞滨节拍化疗开始的中位随访时间为4个月，无患者失访。

### 1.5 统计学方法

采用SPSS 17.0统计软件分析数据，生存分析采用*Kaplan-Meier*法。*P* < 0.05为差异有统计学意义。

## 2 结果

### 2.1 一般情况

中位年龄为67岁（41岁-83岁）。表 1中列出了患者的一般情况。大部分患者是吸烟、腺癌、多线抗肿瘤药物治疗后的。26例患者本次治疗为3线及以上治疗。所有患者均为转移性NSCLC（临床分期为Ⅳ期），且PS评分均在1分及以上。鳞癌患者均未进行表皮生长因子受体（epidermal growth factor receptor, *EGFR*）基因检测，而腺癌患者均为*EGFR*敏感基因无突变者。

**1:** 患者一般情况 Patients'clinical characteristics

Index	*n* (%)
Age (yr)	
≤60	8 (30)
> 60	18 (70)
Gender	
Male	18 (70)
Female	8 (30)
Pathologic type	
Adenocarcinoma	14 (54)
Squamous carcinoma	11 (42)
Adenosquamous carcinoma	1 (4)
Smoking status	
Smoking	17 (65)
Non-smoking	9 (35)
Treatment lines	
3	13 (50)
> 3	13 (50)
Performance status	
1	13 (50)
2	13 (50)

### 2.2 生存结果

入组的26例患者均完成了既定治疗。中位随访时间为4（2-12）个月。患者的中位治疗周期数为2（1-8）个周期，无患者因不良反应停止治疗或减量治疗。无患者达到CR，2例（8%）患者达PR，11例（42%）达SD，13例（50%）为PD。患者的ORR为8%，DCR为50%。生存分析结果显示全部患者的中位PFS为2.0个月。对影响PFS的各因素进行单因素分析中，PS评分为1分的患者优于2分患者（*P*=0.012）（图 1），性别（*P*=0.101）、年龄（*P*=0.330）、吸烟状态（*P*=0.181）、病理类型（*P*=0.881）均没有PFS的统计学差异。

**1:**
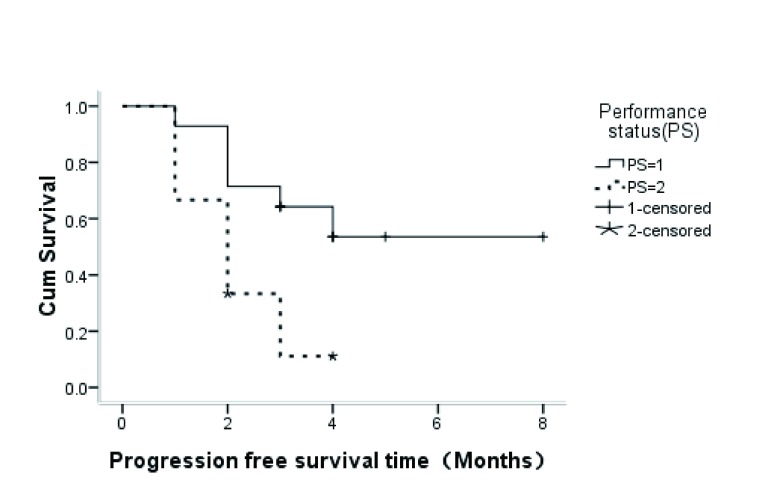
不同体力状态评分患者的无进展生存时间曲线 *Kaplan-Meier* curves for progression-free survival with different performance status

### 2.3 不良反应

26例患者中，大部分患者所出现的副作用为Ⅰ级-Ⅱ级，没有出现Ⅳ级或不可耐受的毒性反应（表 2）。2例患者出现Ⅲ级中性粒细胞减少，给予粒细胞集落刺激因子治疗3 d后缓解，继续治疗。没有患者因AEs出现死亡或因治疗AEs而需要住院治疗。没有患者因AEs而终止治疗。

**2:** 各级以及3级治疗相关AEs（*n*=26） Treatment related adverse events (AEs) with all grades and grade 3 (*n*=26)

AEs	All grades [*n* (%)]	Grade 3 [*n* (%)]
Non-hematological		
Fatigue Nausea Vomiting Diarrhea	5 (19) 8 (31) 3 (12) 1 (4)	0 0 0 0
Hematological		
Anemia Neutropenia	17 (65) 11 (42)	0 2 (8)

## 3 讨论

化疗仍然是晚期NSCLC治疗的基石。虽然多项临床研究结果表明长春瑞滨联合顺铂方案治疗晚期NSCLC的疗效与其他含铂双药方案的疗效相似^[11, 12]^；然而现在临床上长春瑞滨的使用率明显少于其他三代化疗药，主要因为其静脉制剂对血管刺激性大以及严重的白细胞减少发生率高；而长春瑞滨口服制剂可以解决上述问题。对于多线治疗后，PS评分差的晚期NSCLC患者来说，副作用小的单药化疗可能是一种更合适的选择^[13]^。因此，长春瑞滨口服剂型可能是这部分患者的更好选择。

MOVE研究^[14]^观察了43例70岁以上的晚期NSCLC患者接受一线节拍长春瑞滨口服治疗的研究，1例患者达CR，7例患者达PR，ORR为18.6%，17例患者SD时间超过3个月，总DCR为58.1%。中位PFS为5（2-21）个月，总生存时间为9（3-29）个月。我们的研究中ORR为8%，DCR为50%，中位PFS为2.0个月。我们研究中ORR和DCR低，考虑原因为96%的患者接受过3线以上治疗，多线治疗效果均差于一线治疗结果。一项三线化疗的回顾性研究^[15]^结果发现，一线治疗的ORR为38%，二线和三线治疗的ORR分别仅为14%和6%。这与我们研究的ORR结果相近。

德国Guetz等^[16]^也在晚期NSCLC患者中进行了节拍长春瑞滨的剂量爬坡研究，采用每天20 mg-50 mg，治疗三周停一周的方法。30 mg剂量组中，患者的耐受性良好。40 mg剂量组中，5例患者中有2例出现剂量限制性毒性（dose-limiting toxicities, DLTs），50 mg剂量组中，6例患者中有3例出现DLTs。最终研究推荐的用法是第一周期化疗采用每天30 mg，第2周期化疗采用每天40 mg。我们研究中均为中国人，且多程治疗后PS评分高，因此我们的剂量以及口服频率要低于该研究。采用30 mg每周3天的连续治疗，耐受性好。

MOVE研究^[14]^中贫血发生率为44.0%，乏力发生率为32.4%，腹泻发生率为10.5%。治疗没有对患者生活质量产生影响。我们研究中可以看到，患者的血液学毒性发生率高于非血液学毒性，贫血发生率为65.4%，高于MOVE研究的数据，考虑与我们研究中入组患者大多为3线及以上治疗的患者、多程治疗后患者的骨髓功能较差有关。但整体来说，该治疗安全性高，无患者因AEs需要住院治疗。

虽然我们研究的随访时间不够长，患者数量小，但我们的研究结果已经发现对于多程治疗后的晚期NSCLC患者来说，口服长春瑞滨节拍化疗可作为治疗晚期NSCLC，尤其是PS评分差的患者的有效治疗，服用方便，安全性高。而对于口服长春瑞滨节拍化疗的应用剂量和频率还需要进一步研究。
